# Data on the awareness and adoption of ICT in town planning firms in Lagos state, Nigeria

**DOI:** 10.1016/j.dib.2018.08.036

**Published:** 2018-08-17

**Authors:** Adedotun O. Akinola, Taofik Salau, Adedapo Oluwatayo, Oluwatosin Babalola, Hilary I. Okagbue

**Affiliations:** a Department of Architecture, Covenant University, Ota, Nigeria; b Department of Urban and Regional Planning, University of Lagos, Akoka, Nigeria; c Department of Mathematics, Covenant University, Ota, Nigeria

**Keywords:** ICT, Autocad, GIS, Likert, Awareness and adoption, Survey analytics, Town planning, Statistics

## Abstract

This dataset covers an investigation into awareness and adoption of information and communication technology (ICT) in town planning firms in Lagos state, Nigeria. A survey of thirty (30) town planning firms in Lagos state, Nigeria was conducted. The survey was carried out between January to March 2017 by the use of questionnaires. The dataset contains responses on the factors that influence ICT usage, barriers and constraints of ICT usage. The five (5) point Likert scale was used for quantitative data analysis. The data can help identify the level of ICT usage, identify areas of concern and solutions can be proffered based on the results of the analysis.

**Specifications Table**

**Table:** 

Subject area	*Construction and Building*
More specific subject area	*Urban and Regional planning*
Type of data	*Tables and Figures*
How data was acquired	*Field Survey*
Data format	*Raw and analyzed*
Experimental factors	*Cross-sectional survey research design of town planning firms to determine factors that influence ICT usage, barriers and constraints of ICT usage.*
Experimental features	*Multistage sampling selection, simple boxplot, stacked bars, correlation matrix and analysis of variance (ANOVA)*
Data source location	*Lagos, Nigeria*
Data accessibility	*All the data are in this data article*

**Value of the data**

• The dataset can also be used by professional bodies in organizing training program seminars and workshops for Town planners in Lagos, Nigeria.
• The data could be used to advocate ICT usage for professional bodies.
• The data can be used for educational and research purposes [1].
• The questionnaire can be adapted, adopted for a similar research on this subject.

## 1 Data

The article describes data obtained from town planners in different town planning firms on their awareness and adoption of ICT. The data were mainly the analysis of responses from administering questionnaires. A total of 39 questionnaires was administered among the town planning firms in Lagos state, out of which only 30 questionnaires (70%) were retrieved for analysis. The questionnaire can be assessed as Supplementary data. Data collected using the questionnaire was analyzed and that provided the study information. Descriptive statistics (univariate analysis) using mean, frequency, percentages and proportions were used in the data analysis. The five point Likert scale was adopted to facilitate the data analysis. The data presented are the socio-demographics of the respondents and the organizational characteristics of the firm which are the variables used to measure the level of awareness and adoption of ICT in the surveyed Town planning firms.

### 1.1 Socio-demographics of the respondents

**Fig. 1:**
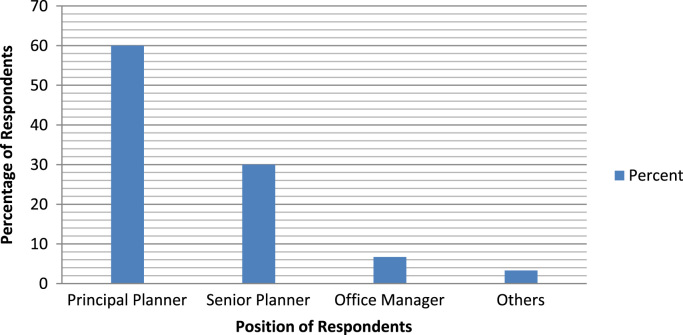
Position of respondent in firm.

**Fig. 2:**
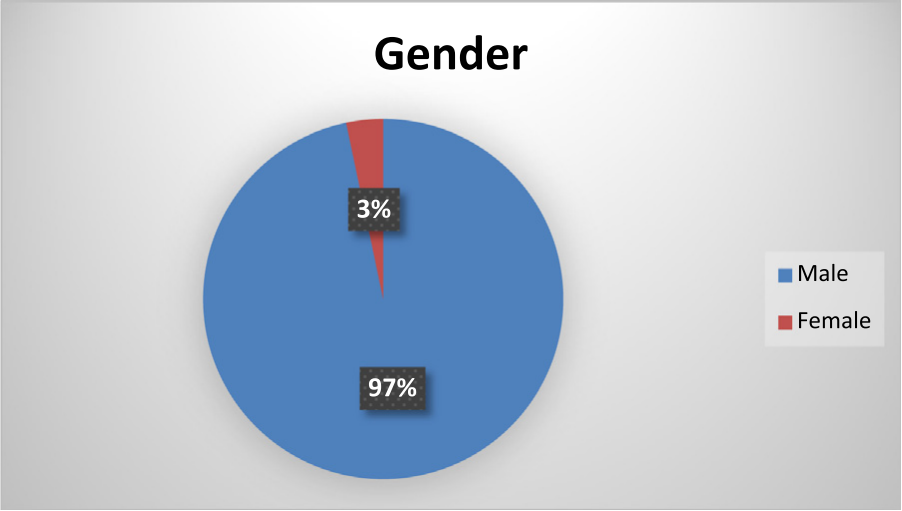
Genders of respondents.

**Fig. 3:**
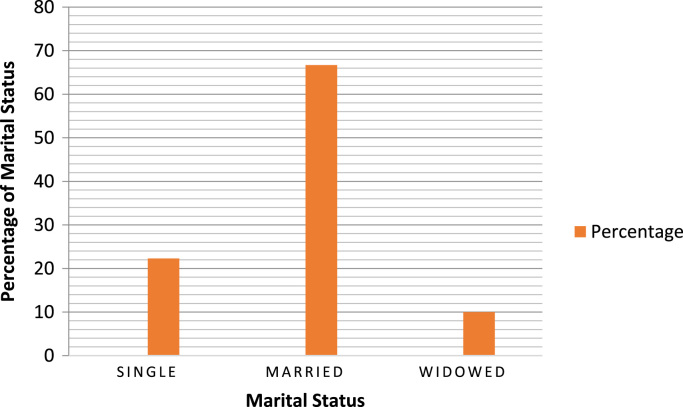
Marital status of respondents.

**Fig. 4:**
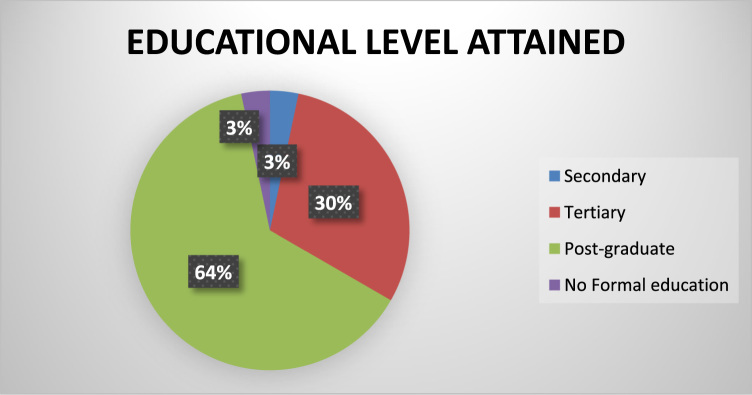
Educational Level of respondents.

**Fig. 5:**
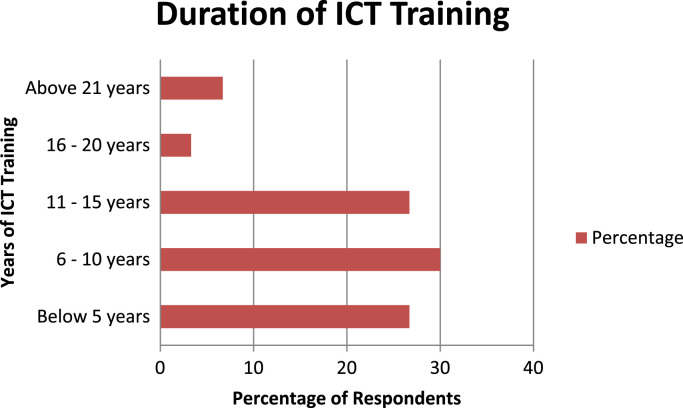
Duration of ICT training of the respondents.

The socio-demographics are presented in percentages to facilitate easy comparison and interpretation. These are presented as follows: position of the respondents in the respective Town planning firms (Fig. 1), gender of the respondents (Fig. 2), age of respondents (Table 1), marital status of respondents (Fig. 3), educational level of respondents (Fig. 4), years of experience (Table 2) and duration of ICT training of the respondents (Fig. 5).

**Table 1:** Age of respondents.

Age of respondents	Percentage
Below 25	6.7
25–40 yrs	43.3
41–50 yrs	23.3
61–70	26.7
Total	100.0

**Table 2:** Years of experience of the respondents.

Years of experience	Percentage
Below 5 years	13.3
6–10 years	23.3
11–15 years	33.3
16–20 years	13.3
Above 21years	16.7
Total	100.0

### 1.2 Organizational characteristics of the firms surveyed

**Fig. 6:**
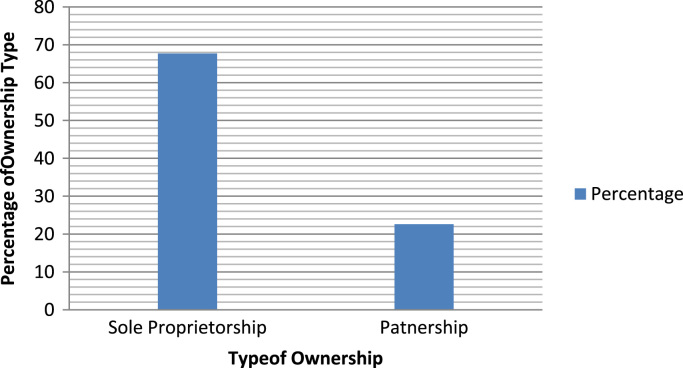
Ownership of the surveyed firms.

**Fig. 7:**
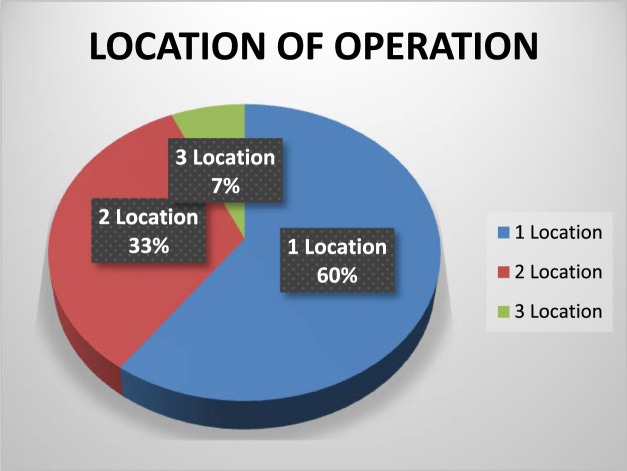
Numbers of locations of operations of the surveyed firms.

**Fig. 8:**
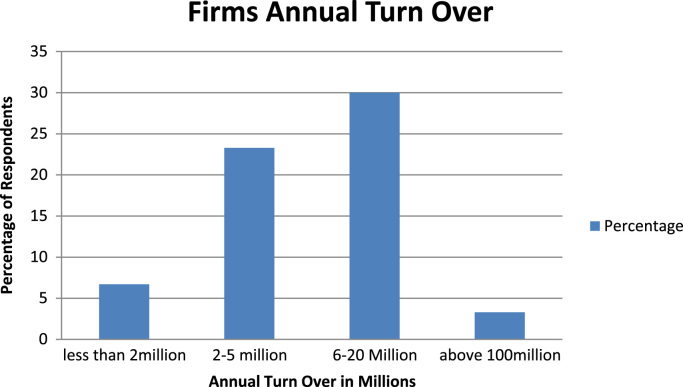
Surveyed firms annual turnover in millions of Naira.

**Fig. 9:**
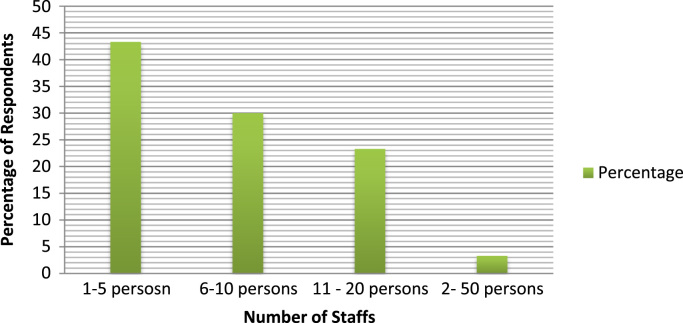
Number of staffs in the surveyed firms.

**Fig. 10:**
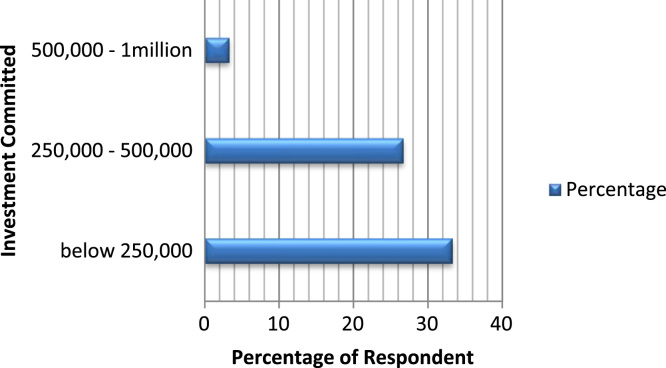
Investment committed annually to ICT by the surveyed firms.

**Fig. 11:**
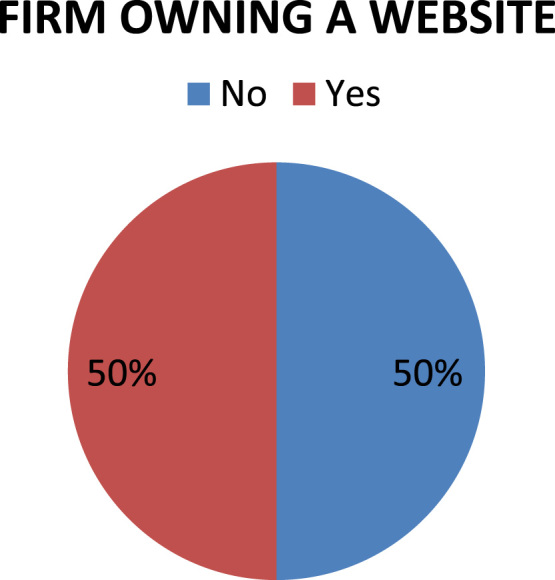
Ownership of website by the surveyed firms.

**Fig. 12:**
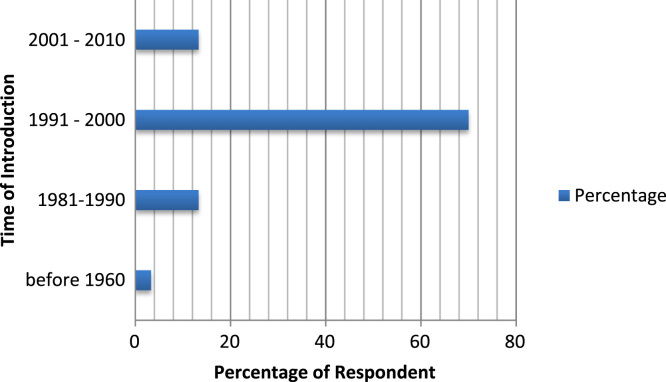
Time of introduction to ICT in the surveyed firms.

The organizational characteristics of the firm are vital in deterring the extent of the firms ‘awareness and adoption of ICT. These are presented as follows: ownership of the surveyed firms (Fig. 6), numbers of locations of operations of the surveyed firms (Fig. 7), year of establishment of the surveyed firms (Table 3), surveyed firms’ annual turnover in millions of Naira (Fig. 8), number of staffs in the surveyed firms (Fig. 9), program for training your staff in ICT applications within the firm (Table 4) and training form of ICT literates (Table 5). Others are: investment committed annually to ICT by the surveyed firms (Fig. 10), ownership of the websites of the surveyed firms (Fig. 11), time of introduction to ICT in the surveyed firms (Fig. 12), workstations operated by the surveyed firms (Table 6), internet connectivity of the surveyed firms (Table 7), facilities available in the surveyed firms (Table 8) and ease of change of system and applications at the surveyed firms (Table 9).

**Table 3:** Year of establishment of the surveyed firms.

Year of establishment	Percentage
1961–1970	0.0
1971–1980	10.0
1981–1990	6.7
1991 or above	83.3
Total	100.0

**Table 4:** Program for training your staff in ICT applications within the firm.

Response	Percentage
Yes	26.7
No	73.3
Total	100

**Table 5:** Training form of ICT literates of the surveyed firms.

Training form	Percentage
One-on-one	20.0
Launch and learn	16.7
Did-it-yourself	13.3
Classroom	10
Non response	40
Total	100

**Table 6:** Work stations operated by the surveyed firms.

Work stations operated	Frequency	Percentage
1–5	20	66.7
6–10	6	20.0
11–20	4	13.3
Total	30	100.0

**Table 7:** Internet connectivity of the surveyed firms.

Internet connectivity	Percentage
No	3.3
Yes	93.3
Total	100.0

**Table 8:** Facilities available in the surveyed firms.

Facilities available in firm	Percentage
Internet and intranet	46.7
Internet, intranet, extranet	20.0
Internet, intranet, extranet, and CSCW	6.7
None of the above	13.3
Unanswered	13.3
Total	100

**Table 9:** Ease of change of system and applications at the surveyed firms.

Ease of change of system and applications	Percentage
Strongly disagree	3.3
Disagree	3.3
Undecided	13.3
Agree	56.8
Strongly Agree	23.3
Total	100.0

## 2 Experimental design, materials and methods

A combination of quantitative and qualitative research method was adopted for this work. A cross-sectional survey was conducted, and in-depth interviews were also conducted to complement the empirical data generated. The data that emanate from the interviews were not included in this work. The questionnaire used in data collection was designed by the researchers and had three sections according to the major research issues addressed in this study. Similar methods and contributions can be seen in [1], [2], [3], [4], [5], [6], [7], [8], [9], [10], [11], [12], [13], [14], [15]. Specifically, the data presented in the article can be helpful in policy implementation and monitoring in ICT adoption and assessing the gains accruable to ICT investments, some of the articles [16], [17], [18], [19], [20], [21], [22], [23], [24], [25], [26], [27], [28], [29], [30], [31], [32] addressed similar issues. In addition, different analytical paths can be explored [33], [34], [35], [36].

Section 1 is centered on the respondents’ personal characteristics (age, sex, marital status, income, education, level of experience, duration of ICT training). Section 2 focused on the organization/facility characteristics of the firm such as; number of branches, the year of the establishment of the firm, the firm׳s annual turnover, the number of staffs in the firm, training programs, ownership of website(s), internet connectivity and perceived number of ICT literates. Section 3 is mainly on ICT usage, responses were solicited from the respondents based on a five point Likert scale: “1” for not at all; “2” for rarely; “3” averagely; “4” for often and “5” for daily. Respondents were also asked to rate the factors that determine the level of ICT usage by their firm on a five point Likert scale: “1” for not at all; “2” not very much; “3” A little; “4” very much and “5” for A great extent, respondents were also asked to rate the level of ICT application usage by your firm on a five point Likert scale: “1” low; “2” Below Average; “3” Average; “4” Above Average and “5” for high. Section 4 is on the benefits of ICT usage on a five point Likert scale: “1” for not at all; “2” not very much; “3” A little; “4” very much and “5” A great extent. Section 5 is on the constraints to ICT usage, respondents were asked to rate the constraints to the use of ICT by their firm on a five point Likert scale: “1” for not at all; “2” not very much; “3” A little; “4” very much and “5” A great extent. Then, Likert scale was used to rank the variables using the sum of the weighted values (SWV) and summarized as the respondents’ index.

### 2.1 Analysis of level of adoption of ICT usage

The following data presented are the measures of the level of adoption of ICT usage in the surveyed firms. These include: design technologies (Table 10), level of usage of word processing, analysis and presentation tools by the firms (Table 11), applications for communication system used by the firms (Table 12), perceived usage of hardware systems as responded by the firms (Table 13), tasks and services performed using ICT in the firms (Table 14), perceived factors that determine the use of ICT by the firms (Table 15), perceived benefits of ICT by the firms (Table 16) and perceived constraints to ICT usage and adoption by the firms (Table 17).

**Table 10:** Representation of the applications for design technologies of the firms.

**Applications**	**L**	**BA**	**A**	**AA**	**H**	**SWV**	**Index**	**Rank**
Autocad	1	0	0	2	26	139	4.63	1ST
ArcGis	3	2	5	5	14	114	3.80	2ND
Autodesk Land development	6	1	4	9	9	101	3.36	3RD
Surfer	8	2	5	5	8	99	3.3	4TH
Sketchup	9	1	3	5	9	91	3.03	5TH
Others	6	4	6	6	6	86	2.86	6TH

L = low, BA = below average, A = average, AA = above average, H = high.

**Table 11:** Level of usage of word processing, analysis and presentation tools by the firms.

**Applications**	**L**	**BA**	**A**	**AA**	**H**	**SWV**	**Index**	**Rank**
MS Word	2	0	0	0	28	149	4.96	1ST
MS Excel	1	0	1	5	22	134	4.46	2ND
MS PowerPoint	3	1	3	5	15	109	3.63	3RD
SPSS	7	0	4	6	10	93	3.1	4TH
Adobe PageMaker	5	2	4	4	11	82	2.73	5TH
Corel draw	6	4	6	6	6	86	2.86	6TH
In-design	9	0	4	7	4	69	2.3	6TH
Illustrator	9	1	3	7	4	68	2.3	7TH
MS Perfect	7	0	4	6	10	93	3.1	8TH

L = low, BA = below average, A = average, AA = above average, H = high.

**Table 12:** Applications for communication system used by the firms.

**Applications**	**L**	**BA**	**A**	**AA**	**H**	**SWV**	**Index**	**Rank**
Internet	1	0	0	2	25	84	2.8	1ST
Video conferencing	11	0	1	5	8	66	2.2	2ND
Electronic data management	8	0	1	6	8	75	2.5	3RD
Intranet	11	1	3	3	7	69	2.3	4TH
Voicemail	11	2	2	2	3	44	1.5	5TH

L = low, BA = below average, A = average, AA = above average, H = high.

**Table 13:** Perceived usage of hardware systems as responded by the firms.

**Applications**	**L**	**BA**	**A**	**AA**	**H**	**SWV**	**Index**	**Rank**
Computer System	0	0	0	4	24	128	4.26	1ST
Printer	0	0	0	4	22	118	3.93	2ND
Plotter	1	1	4	4	17	106	3.53	3RD
GPs	1	1	7	4	13	105	3.50	4TH
Lidar Camera	9	1	3	3	6	50	1.6	6TH
Drone	10	2	3	3	4	55	1.83	5TH

**Table 14:** Tasks and services performed using ICT in the firms.

**Tasks**	**NA**	**RLY**	**AVG**	**OFT**	**DLY**	**SWV**	**Index**	**Rank**
Detailed layout design	0	0	5	14	11	126	4.2	1ST
Report Writing	0	1	1	14	13	126	4.2	2ND
General office Administration	1	0	3	8	17	127	4.2	3RD
Data analysis	0	1	5	17	7	120	4.0	4TH
Presentation works	0	1	5	17	7	120	4.0	4TH
Project planning and management	1	2	4	13	10	119	4.0	5TH
Collaborative works	0	1	6	10	12	120	4.0	6TH
Design/Research Info Search	0	3	4	11	11	117	3.9	7TH
Physical modeling	1	6	3	9	10	108	3.6	8TH
Digital modeling	1	5	9	9	6	104	3.4	9TH
Public relations	3	9	8	2	5	78	2.6	10TH
Others	0	0	0	3	4	32	1.1	11TH

NA = Not at all, RLY = Rarely, AVG = Averagely, OFT = Often, DLY = Daily.

**Table 15:** Perceived Factors that determine the use of ICT by the Firms.

**Factors**	**NAA**	**NVM**	**AL**	**VM**	**AGE**	**SWV**	**Index**	**Rank**
Level of competition	0	1	3	9	16	127	4.2	1ST
Changing trends in global construction	0	1	3	8	16	123	4.1	2ND
Construction industry demands	1	0	2	10	15	122	4.1	3RD
Client/customer demand	1	1	4	5	17	120	4.0	4TH
Job/Project requirement	1	3	11	12	0	100	3.3	5TH

NAA = Not at all, NVM = Not very much, AL = A little, VM = Very much, AGE = A great extent.

**Table 16:** Perceived benefits of ICT by the firms.

**Benefits**	**NAA**	**NVM**	**AL**	**VM**	**AGA**	**SWV**	**Index**	**Rank**
Enhances productivity	1	0	0	0	28	141	4.7	1ST
Saves time	1	0	0	0	28	141	4.7	1ST
Improves public image of user	1	0	0	1	27	140	4.66	3RD
Gives users competitive advantage	1	0	0	1	27	140	4.66	3rD
Facilitates decision making	0	1	1	6	22	139	4.63	5th
Savings in operating cost	1	0	0	4	24	137	4.56	6TH
Makes professional job easier	0	0	0	2	25	133	4.43	7TH
Improves document presentation	0	0	0	0	4	20	0.66	8TH

NAA = Not at all, NVM = Not very much, AL = A little, VM = Very much, AGE = A great extent.

**Table 17:** Perceived constraints to ICT usage and adoption by the firms.

**Constraints**	**NAA**	**NVM**	**AL**	**VM**	**AGA**	**SWV**	**Index**	**Rank**
High cost of professional to employ	2	4	5	4	14	115	3.83	1ST
Inadequate power supply	5	0	5	7	13	113	3.76	2ND
High cost of hardware and software	3	2	7	6	11	105	3.50	3RD
Continual need to upgrade	3	3	8	9	6	99	3.33	4TH
System and computer malfunction	3	4	4	8	10	97	3.23	5TH
Poor security and privacy	6	7	6	4	6	94	3.13	6TH
Incompatibility in software packages	6	4	6	6	7	91	3.03	7TH
Inadequate ICT content in construction	5	4	8	6	6	91	3.03	7TH
Scarcity of professional software	4	10	5	7	3	82	2.93	9TH
Job size and fees not enough for ICT	4	5	8	7	4	86	2.86	10TH
Poor return on investment	7	5	9	3	5	81	2.70	11TH
Personal abuse	8	9	7	2	2	65	2.20	12TH
ICT making town planners redundant	13	5	4	4	3	66	2.16	13TH

NAA = Not at all, NVM = Not very much, AL = A little, VM = Very much, AGE = A great extent.
