# Supplementary material for: Data on the awareness and adoption of ICT in town planning firms in Lagos state, Nigeria
Source: Data Brief. 2018 Aug 17;20:436–47. doi: 10.1016/j.dib.2018.08.036 (PMC6116422; doi:10.1016/j.dib.2018.08.036)
Supplement: Supplementary file 2 — Supplementary Data. [file mmc2.docx]

**QUESTIONNAIRE**

**DEPARTMENT OF URBAN AND REGIONAL PLANNING**

**FACULTY OF ENVIRONMENTAL SCIENCE**

**UNIVERSITY OF LAGOS, NIGERIA**

Dear Sir/ Madam

**INFORMATION & COMMUNICATION TECHNOLOGY (ICT) AND TOWN PLANNING PRACTICES IN PRIVATE FIRMS IN LAGOS STATE**

Kindly give candid answers to the questions below. The questionnaire is designed to collect information for my professional Masters in Urban and regional planning(MURP) research on investigating the level of usage Information and Communication Technology (ICT) in private town planning firms in Lagos State with a view to developing strategies that can strengthen the existing state of adoption/Usage.

I would be grateful if the principal or a senior town planner completes the questionnaire. The goal of this research is to understand the level of adoption of ICT in town planning firms Nigeria with particular focus on the design and administration packages (software and hardware ) as used in practice. Please be assured that the information provided by you will be treated in strict confidence and the results will be published only in an aggregated form. Your firm will remain anonymous. I hope to give you a summary of the findings at the end of the research

Yours sincerely

Akinola Adedotun

dtn_akin@yahoo.com

**A BACKGROUND INFORMATION OF RESPONDENT**

1. Name and address of your firm…………………………………………………………..

E-mail (firm)………………………..Website (firm)…………………………………….

1. What is your position in the firm? Principal town planner ( ) Senior town planner( ) office manager( ) others( )
2. What is your gender?

( ) male ( ) female

1. How old are you? Below 25 years( ), 25 -40( ), 41-50(3), 51-60(4). 61-70(5), 71- above ( )
2. What is your marital status?

( ) single ( ) married ( ) widowed ( ) Divorced ( )Separated

1. What is the highest level of education you have completed?

( ) secondary ( ) tertiary ( ) post-graduate ( ) No formal education

1. What is your level of experience(years)

( ) <5 ( ) 6-10 ( ) 11-15 ( ) 16-20 ( )>21

1. What is your duration of ICT training(months)

( ) <5 ( ) 6-10 ( ) 11-15 ( ) 16-20 ( )>21

**B ORGANISATIONAL/FACILITIES CHARACTERISTICS OF THE FIRM**

1. From how many locations (branches) do you operate? ................. ( 1 ) , ( 2 ), ( 3 ), ( 4 ) ,( 5 )
2. What year was the firm established ?........................................before 1961-1970( ) 1971-1980( ) 1981-1990( ) 1991-2000( ) 2001-2010( ) 2011-2017( )
3. What is the firm’s annual turnover………………………………… less than N2 million( ) N2 million –N5 MILLION( ) N6 million –N20 million ( ) N21 million – N100 million( ) above N1OO million ( )
4. How many staffs work in your firm? .......................1-5 persons ( ) 6-10 persons( ) 11-20( ) 20 -50 persons( ) 51 and above( )
5. How many persons have town planning training?............... 1-5 persons( ) 6-10 persons( ) 11-20( ) 20 -50 persons( ) 51 and above( )
6. How many persons are ICT Literate? None ( ) 1-5 persons ( ) 6-10 persons ( ) 11-20( ) 20 -50 persons( ) proficiency level
7. Is there an existing programme for training your staff in ICT Applications within the firm? No ( ) yes( ) undecided( )
8. If yes in above what form?

Classroom format ( ) one-on –one( ) launch and Learn( ) do –it -yourself( )

1. How much investment is committed annually to the training programme? Less than N250,000 ( ) N250,000-N500000( ) N500,000-N1 million ( ) N1 million -5 million( ) above N5 million ( )
2. Do your firm have a Website? NO( ) Yes( ) Undecided( )
3. When did your firm first introduce computers for operations or transit from manual design to CAD………………………………… before 1961-1970( ) 1971-1980( ) 1981-1990( ) 1991-2000( ) 2001-2010( ) 2011-2017( )
4. How many workstations do you operate…………………..1-5( ) 6-10( ) 11-20( ) 20-50( ) 51 and above( )
5. Do you have internet connectivity? No( ) Yes( ) Undecided( )
6. Which of these facilities are available in your firm ?Internet( ) internet and intranet( ) internet, intranet , extranet( ) internet, intranet , extranet and computer supported Collaborative Work(CSCW)( ) none of the above( )
7. When changes emerge in the industry, is it easy to change systems and applications in your firm?

Strongly disagree ( ) disagree ( ) undecided ( ) Agree( ) strongly agree( )

C **LEVEL OF ADOPTION OF ICT USAGE**

1. **Which of the following Tasks are being performed in your firm and at what frequency?**

| Task/Services | Not at all | Rarely | Averagely | often | Daily |
| --- | --- | --- | --- | --- | --- |
| Detailed layout design |  |  |  |  |  |
| Data Analysis |  |  |  |  |  |
| Project planning and management |  |  |  |  |  |
| Physical modelling |  |  |  |  |  |
| Digital Modelling |  |  |  |  |  |
| Presentation Works |  |  |  |  |  |
| Collaborative Work |  |  |  |  |  |
| General office Administration |  |  |  |  |  |
| Report Writing |  |  |  |  |  |
| Public relations |  |  |  |  |  |
| Design/Research Info Search |  |  |  |  |  |
| Others |  |  |  |  |  |

1. **Kindly rate the factors that determine ICT usage by your firm. Note: 5=A great extent, 4= very much, 3=A little, 2=not very much. 1= not at all**

| FACTORS DETERMINE USAGE OF ICT | 1 | 2 | 3 | 4 | 5 |
| --- | --- | --- | --- | --- | --- |
| Changing trends in global construction |  |  |  |  |  |
| Client/ customer demand |  |  |  |  |  |
| Construction industry demands |  |  |  |  |  |
| Job/project requirement |  |  |  |  |  |
| Level of competition |  |  |  |  |  |

1. **Kindly indicate the level of ICT usage by your firm. Note: 5=high , 4= Above Average,3=Average, 2=Below Average. 1=low**

| **Design technologies** | 1 | 2 | 3 | 4 | 5 |
| --- | --- | --- | --- | --- | --- |
| AutoCad |  |  |  |  |  |
| ArcGis |  |  |  |  |  |
| Autodesk Land Development |  |  |  |  |  |
| Autodesk 3Ds Max |  |  |  |  |  |
| Surfer |  |  |  |  |  |
| Sketchup |  |  |  |  |  |
| Other(please indicate) |  |  |  |  |  |
| **Word processing , Analysis and presentation tools** | | | | | |
| Ms Word |  |  |  |  |  |
| Ms Perfect |  |  |  |  |  |
| Ms Excel |  |  |  |  |  |
| Ms Powerpoint |  |  |  |  |  |
| Adobe Pagemaker |  |  |  |  |  |
| Corel draw |  |  |  |  |  |
| In-design |  |  |  |  |  |
| Illustrator |  |  |  |  |  |
| SPSS |  |  |  |  |  |
| **Communication system** | | | | | |
| Internet |  |  |  |  |  |
| Intranet |  |  |  |  |  |
| Voicemail |  |  |  |  |  |
| Electronic data management(EDM) |  |  |  |  |  |
| Video conferencing |  |  |  |  |  |
| **Hardware** | | | | | |
| Computer system |  |  |  |  |  |
| Printer |  |  |  |  |  |
| Plotter |  |  |  |  |  |
| GPS |  |  |  |  |  |
| Lidar Camera |  |  |  |  |  |
| Drone |  |  |  |  |  |
| Others( please indicate) |  |  |  |  |  |

D **BENEFITS OF ICT USAGE**

1. **Kindly rate the benefits to the use of ICT by your firm. Note: 5=A great extent, 4=very much ,3=A little , 2= not very much, 1=not at all**

| Benefits of Ict usage to the consultants | 1 | 2 | 3 | 4 | 5 |
| --- | --- | --- | --- | --- | --- |
| Makes professional job easier |  |  |  |  |  |
| Facilitates decision making |  |  |  |  |  |
| Savings in operating costs |  |  |  |  |  |
| Improves public image of the user |  |  |  |  |  |
| Gives users competitive advantage |  |  |  |  |  |
| Enhances productivity |  |  |  |  |  |
| Saves time |  |  |  |  |  |
| Improves document presentation |  |  |  |  |  |
| Others |  |  |  |  |  |

**E CONSTRAINTS TO ICT USAGE**

1. **Kindly rate the factors that constraint s to the use of ICT by your firm. Note: 5=A great extent, 4=very much ,3=A little , 2= not very much, 1=not at all**

| Constraints to the use of Ict by the consultants | 1 | 2 | 3 | 4 | 5 |
| --- | --- | --- | --- | --- | --- |
| Continual need to upgrade |  |  |  |  |  |
| Job size and fees not enough for ICT |  |  |  |  |  |
| Personal abuse |  |  |  |  |  |
| High cost of hardware and software |  |  |  |  |  |
| Poor security and privacy |  |  |  |  |  |
| High cost of professional to employ |  |  |  |  |  |
| System and computer malfunction and virus attacks |  |  |  |  |  |
| Incompatibility in software packages |  |  |  |  |  |
| Inadequate ICT content in construction education |  |  |  |  |  |
| Scarcity of professional  software |  |  |  |  |  |
| Poor return on investment |  |  |  |  |  |
| Inadequate power supply |  |  |  |  |  |
| ICT making professsionals redundant |  |  |  |  |  |
|  |  |  |  |  |  |
